# Traumatic Arteriovenous Fistula After Kickboxing Injury: A Case Report and Review of the Literature

**DOI:** 10.5812/atr.15575

**Published:** 2014-03-20

**Authors:** Masoud Rezvani

**Affiliations:** 1 Department of Surgery, Abington Memorial Hospital, Abington, Pennsylvania, USA

**Keywords:** Arteriovenous Fistula, Wound Healing, Trauma Centers

## Abstract

**Introduction:**

A traumatic arteriovenous fistula (AVF) after repetitive blunt trauma has not been described previously. In a 34-year-old male, the first reported case of such an injury after repetitive blunt trauma is described.

**Case Presentation:**

A 34-year-old gentleman presented with a non-healing ulcer near his medial malleolus. A bone scan was performed and then treated for presumed osteomyelitis. An arteriogram confirmed an AVF, and coil embolization was performed with complete occlusion of the AVF. Subsequently, the ulcer healed rapidly with no complication. Along with the cause of AVF, this case is notable for symptom presentation.

**Conclusions:**

Arteriovenous fistula after blunt trauma can present as a non-healing venous stasis ulcer, which could be treated non-invasively.

## 1. Introduction

Arteriovenous fistula (AVF) is a commonly described complication after penetrating trauma and orthopedic injuries, but is rarely seen after blunt trauma. We described the first reported case of a traumatic AVF fistula that resulted from a repetitive kickboxing injury. This patient presented with non-healing venous stasis ulcer in his lower extremity.

## 2. Case Presentation

**Figure 1.**
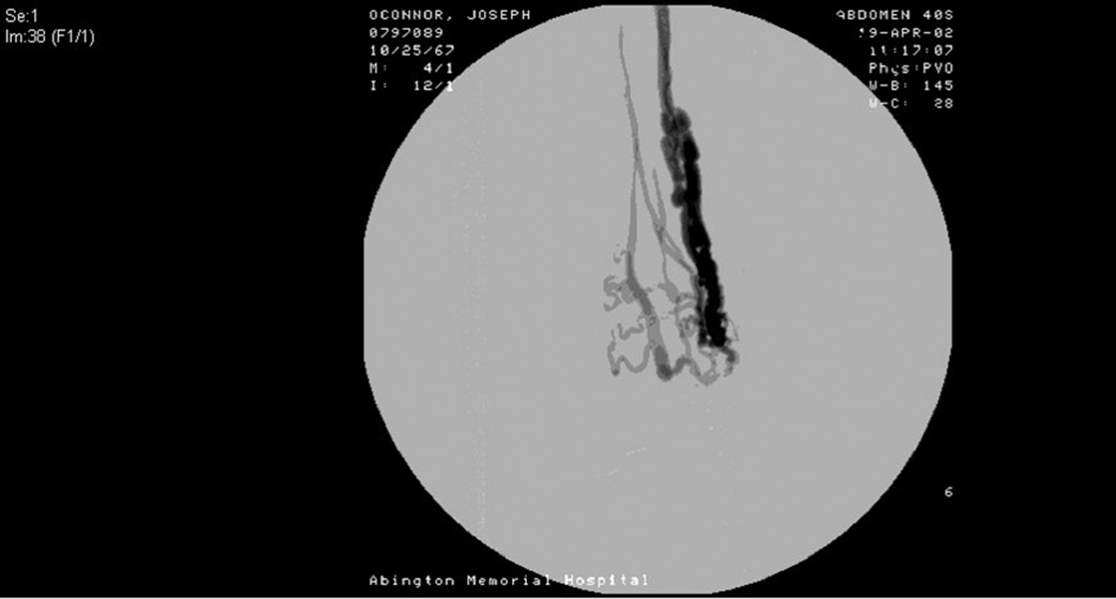
Hypertrophic Anterior Tibial Artery With Direct Connection to Venous Circulation (Arteriovenous Fistula)

**Figure 2.**
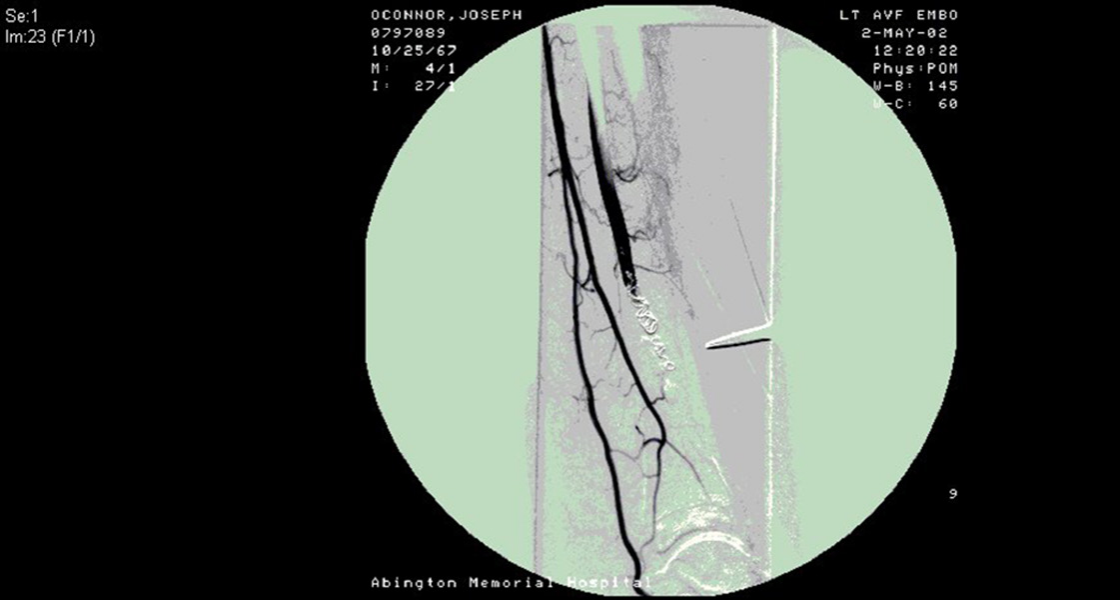
Arteriovenous Fistulas After Embolization With Posterior Run-off to Foot

A 34-year-old male, otherwise healthy, presented with a non-healing ulcer on his left lower extremity of approximately 4 months duration. He noted a 1.5 cm ulcer above the medial aspect of his left ankle, which initially healed, but subsequently reopened. The patient denied rest pain or claudication of his left lower extremity. He noted beating around the ulcer after more than 10 minutes of his leg elevation. History revealed the patient was a competitive kickboxer, and he admitted to repetitive blunt trauma to the anterior portion of his left leg. A bone scan showed increased radionuclide accumulation involving the left lower leg, ankle, and foot. Osteomyelitis was suspected, and he was started on ciprofloxacin, 400 mg every 12 hours. After an incomplete response to the treatment, he was referred for vascular surgery evaluation. The wound was found to have serous drainage and was associated with swelling and palpable thrill over the left ankle. He had palpable dorsalis pedis (DP) and posterior tibialis (PT) pulses. Left lower extremity contrast arteriogram revealed hypertrophy of the left anterior tibial artery (AT) to the level of an arteriovenous fistula approximately 10 cm proximal to the ankle joint (Figure 1). Subsequently, anterior tibial artery was cannulated through femoral and popliteal artery and a 3 mm coil was placed distally to the AVF and 5 mm, 8 mm, and 10 mm coils placed proximally (Figure 2). Run-offs to lower part of the leg and foot were confirmed through tibioperoneal trunk by multiple angiographic views (Figure 2). 

The patient was discharge and instructed to continue antibiotics and local wound care consisting of Papain-Urea (Panafil^R^) cream.

Two weeks later, the ulcer appeared to be healing with granulation tissue. At the patient’s 4-month follow-up, he had palpable PT and dopplerable DP pulse, and there was no evidence of the AVF and wound had healed.

## 3. Discussion

Traumatic arteriovenous fistulas are among the least common vascular injuries with the incidence of 2.3% to 3.9% (1). When an artery is damaged and blood extravasates into a closed anatomical space, a pulsatile hematoma is produced. Organization of its wall causes a false aneurysm. When there is a venous damage in the same region, an arteriovenous fistula is established (2). The most frequency location of traumatic AVF are extremities (43%), head and neck (33%), and visceral (24%) (3). An AVF is a difficult type of injury to diagnose because external hemorrhage may be limited and ischemia may be absent. Most of patients with this type of injury are asymptomatic. The earliest signs of an AVF include bruit and swelling and mostly, bruit is absent initially due to partial occlusion of fistula with thrombosis (3).

Blunt trauma to lower extremity with significant associated orthopedic injuries, including closed or open fractures of tibia or fibula, are well reported as causes of arteriovenous fistula (4, 5). Schwartz and Rankow reported a case of an amateur boxer with a traumatic AVF following repetitive trauma to the right side of his face and was ligated by open operation (6). Keeling et al. reported a case of an AVF of the inferior gluteal artery following blunt buttock trauma that was treated using a combination of surgery and glue embolization by interventional radiology (7). To the best of our knowledge, this is the first case report of blunt trauma to soft tissue, without associated penetrating or orthopedics injury, that caused AVF of anterior tibial artery which resulted in venous hypertension and venous stasis ulcer.

Not all AVFs need immediate intervention, and many resolve spontaneously. If a named blood vessels is involved, it should be treated because of serious adverse effects even in very distal extremities (8). Previous described AT arteriovenous fistula was recommended surgical exploration with ligation of artery and vein above and below the fistula and primary repair of artery and sometimes vein (9). Today, technical advances in interventional radiology made the management of traumatic AVF less invasive and more effective. Some advantages such as using local anesthesia, minimal blood loss and short hospital stay are beneficial and has been described successfully (10). Halabi et al. described a patient following motorcycle accident and closed fracture of tibia who developed an AVF in posterior tibial artery. This patient underwent coil embolization and failed the treatment. Subsequently, the AVF fistula was successfully closed by stent placement (11) Peeters et al. described transvascular balloon embolization technique for treatment of traumatic AVF in radial and peroneal artery successfully (12).

The current case is the first report of traumatic AVF after kickboxing in leg, which presented as a non-healing wound and was treated successfully by coil embolization. Despite the rarity of this complication, surgeons should be aware of possibility of this presentation as a venous stasis wound. Interventional radiology is a very effective and non-invasive approach to treat this complication. However, more studies and follow-up is required to approve the safety and efficacy of this method.
